# Biochar in the British print news media: an analysis of promissory discourse and the creation of expectations about carbon removal

**DOI:** 10.1080/09505431.2023.2285057

**Published:** 2023-11-28

**Authors:** Brigitte Nerlich, Carol Morris, Catherine Price, Holly Harris

**Affiliations:** a Institute for Science and Society, School of Sociology and Social Policy, University of Nottingham, Nottingham, UK; b School of Geography, University of Nottingham, Nottingham, UK; c School of Anthropology and Conservation, University of Kent, Canterbury, UK

**Keywords:** Biochar, greenhouse gas removal, media, promissory discourse, rhetorical analysis, hyberbole

## Abstract

Biochar is amongst a growing suite of approaches developed to address the climate crisis by removing carbon dioxide from the atmosphere; yet public awareness of biochar is low. In this situation, mass-media reporting plays an important role in making an issue public and in creating expectations about its risks and benefits. In British broadsheet newspapers, a promissory, future-oriented discourse on biochar has emerged that is rhetorically configured through, for example, evaluative adjectives, verbs, hyperbole, and allusions to literary and cultural symbols that confer a sense of mystique. Biochar is promoted as an almost magical fix, based on its ability to soak up and store carbon, improve soil health, increase crops yields, and reduce pollutants. Conversely, some of the possible negative aspects of biochar are couched in the form of sarcasm and parody, while others are made invisible. This sets biochar up as a moral good that the public ought to accept, rather than opening up a public debate about its risks and benefits. Engaging in a fine-grained rhetorical analysis of the way promises about biochar are constructed expands the methodological and empirical repertoire of the sociology of expectations and, in future, can be applied to the analysis of other emerging climate change technologies, especially those relating to carbon removal.

## Introduction

**Figure 1.**
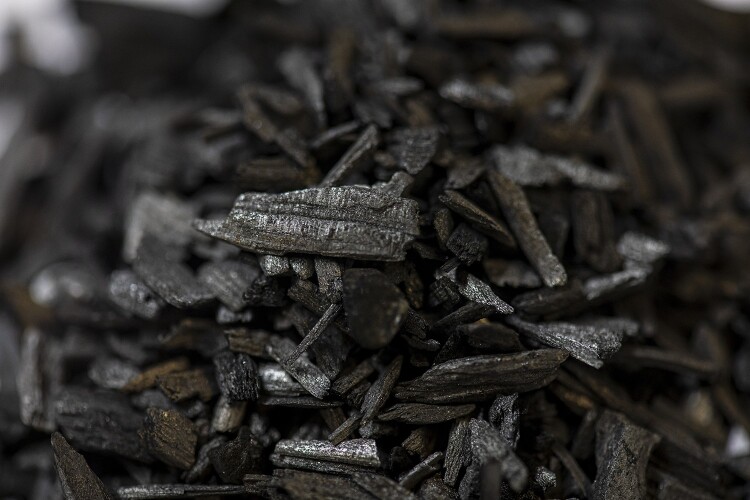
Biochar derived from wood (Photo by the authors).

Biochar is amongst a growing suite of approaches developed to address the climate crisis by removing carbon dioxide from the atmosphere. The term ‘biochar’ is a blend of the words ‘biomass’ and ‘charcoal,’ a lexical compound that was first used in the 1990s (*OED*, online). This carbon-rich substance is typically derived from plant materials including wood and forestry wastes and agricultural crop residues. Other source materials can be animal and human wastes, municipal food waste and invasive plants. It is produced through pyrolysis, a process that entails the thermal decomposition of biomass at high temperatures and under oxygen-deprived conditions (Saxe *et al*., 2019). Biochar is the solid fraction of pyrolysis with bio-oil (liquid) and syngas (gas) the other two by-products. From a greenhouse gas removal (GGR) perspective, biochar needs to be produced and deployed ‘at scale.’ In the UK, the estimated potential of greenhouse gas removal for biochar is 6–41 MtCO_2_ per year, although globally, it is projected to be between 1.9 and 4.8 GtCO_2_ per year (Royal Society and Royal Academy of Engineering, 2018). Biochar is already produced and used on a small scale by individual gardeners, allotment holders and farmers using simple kilns, and can contribute to other environmental objectives such as soil health and woodland management for biodiversity benefit (Figure 1).

Although biochar is attracting significant interest from a range of elite stakeholders, ‘much of the public have not even heard of biochar’ (Nicholas *et al*., 2022, p. 2). In order to make biochar public, and to enable as broad a reflection on and debate around this emerging greenhouse gas removal (GGR) technology as possible, it has to be discussed in the media, including the print news media. This article presents an examination of this coverage. In doing so, it builds on a tradition of other work analysing the media coverage of carbon reduction and storage technologies (Nerlich and Jaspal, 2012, 2013; Porter and Hulme, 2013; Luokkanen *et al*., 2014).

The media constitute an important source of societal information concerning developments in science and technology (Anderson *et al*., 2005). Although traditional print and broadcast media now compete with social media, studying how the print news media represents an emerging technology can provide useful insights into how public views may be shaped and by whom (Harvey, 2022). This is particularly important where emerging technologies, such as biochar, are still relatively unknown to publics (Wright *et al*., 2014; Sweet *et al*., 2021) and views and attitudes can be shaped in one way or another.

The article therefore explores the following questions: How is biochar represented in the British print news media? How do those representations convey specific expectations? These questions are answered by analysing major themes in print news media reportage and their promoters. Given the general expectations identified in our analysis – that biochar promises to have many benefits – we also ask: How is this made worthy of readers’ attention in print news media? This question is answered through an analytical focus on the lexical and rhetorical tools that are used to promote and contest biochar. This contrast is important because the relative balance between the two rhetorical moves may shape public understanding, expectations and perceptions of biochar and societal engagement with this emerging climate technology. A multi-disciplinary approach is developed for this task: linguistic analysis tools are integrated into the sociology of expectations, thereby contributing a novel methodology and findings to the latter.

In the section that follows, we provide further context of biochar before describing our analytical approach. We then analyse a spectrum of rhetorical and lexical tools which are used to structure the promissory discourse of biochar within British broadsheet newspapers, that is, a discourse implying promises of a better future. This rhetoric underpins the public face of biochar in our newspaper corpus and sets the scene for acceptance of this new technology rather than for public debate. We end this article by discussing what these findings mean for future social scientific studies of biochar and the sociology of expectations. We argue that this rhetoric may have important implications for creating expectations not only for biochar but also other carbon removal/carbon capture technologies.

## Background

Biochar is attracting attention from policymakers, scientists and entrepreneurs because it holds, for relatively long periods, a proportion of the carbon from its biomass source material that would otherwise be released to the atmosphere if that material was burned for energy production or left to decompose (Clare *et al*., 2014; Saxe *et al*., 2019). The exact length of time carbon remains in the biochar is contested. The International Biochar Initiative (2022) claims this can happen for 1440–14,500 years, whilst others claim the duration is dependent upon feedstocks and the conversion process (Roberts *et al*., 2010; Hansson *et al*., 2021). In addition to its potential to remove greenhouse gases from the atmosphere, biochar is also said to help improve soil health by increasing water and nutrient holding capacity with associated benefits to agricultural, horticultural and also silvicultural crop yields (Rittl *et al*., 2015; Latawiec *et al*., 2016; Otte and Vik, 2017; Bezerra *et al*., 2019; Pourhashem *et al*., 2019; Saxe *et al*., 2019). Biochar has also been investigated as an animal feed to establish if it has potential to improve livestock health, milk quality and to reduce ammonia emissions from livestock (Innovative Farmers, 2020). Currently biochar’s use in food production contexts, both for GGR and crop yield benefits, is largely experimental. However, the National Farmers Union has identified biochar as one of several approaches that may help the UK agricultural sector achieve net zero by 2040 (NFU, 2021).

**Figure 2.**
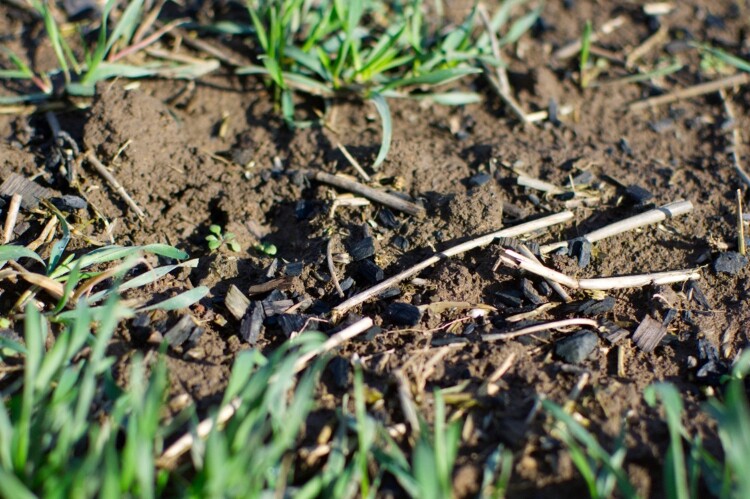
Biochar applied to cropped land (Photos by the authors).

In addition to its deployment in food and forestry production, biochar can also be applied within a variety of other land use contexts such as quarries, embankments and golf courses where its benefits are understood to be carbon sequestration and land restoration (TerrAffix, 2022). Further, biochar is sold to domestic gardeners as a peat substitute (Carbon Gold, 2022). Biochar features in alternative energy production systems such as bioenergy with biochar capture and storage (BEBCS instead of BECCS – bioenergy with carbon capture and storage). It is also seen as a potential additive to road construction materials, or aggregates in cement and concrete production where the interest is to help offset the carbon emissions associated with these industries (Buck, 2019). Biochar can therefore be a waste management strategy, as part of energy production, as well as a material for keeping carbon in soils and a soil enhancer. It is, it seems, a versatile material (Figure 2).

Nevertheless, and in spite of several decades of research, considerable risks and uncertainties remain surrounding biochar’s GGR potential as well as its many claimed side-benefits. Uncertainty surrounds the ability of biochar to sequester carbon over long periods of time once applied to agricultural soils (British Society of Soil Science, 2021). Trade-offs between land requirements for food production and for biochar feedstock production are recognised by the advocacy group Green Alliance (2022), the Royal Society and the Royal Academy of Engineering (2018), and policymakers (Houses of Parliament, 2010). Biochar has also not been demonstrated at scale, so its effectiveness in a range of settings is as yet unproven (Green Alliance, 2019; Committee on Climate Change, 2020). Whilst this is not comprehensive overview of the risks and uncertainties associated with biochar, it provides a flavour of potential issues.

Biochar is one of five approaches to GGR currently funded by UK Research and Innovation (UKRI) under its £30 million GGR demonstrator programme, a major research investment. In May 2021 the biochar demonstrator began, a project involving field trials with arable farmers in England, coordinated by the University of Nottingham (Vaughan, 2022). Such stakeholder engagement is widely recognised as crucial to the assessment of new and emerging sciences and technologies and to the development of responsible research and innovation (Macnaghten and Chilvers, 2014; Bellamy *et al*., 2016, 2017; Chilvers and Kearnes, 2020). The GGR demonstrator projects are designed to inform GGR policy in the UK. The fairly longstanding GGR policy support for afforestation, i.e. the planting of new forests (Schenuit *et al*., 2021), could be seen as an indirect form of policy support for biochar as trees are one type of biochar feedstock. However, there is no public policy that currently directly incentivises the production and use of biochar. This may, in part, explain the lack of attention given to support measures in the reporting.

In theory, biochar’s use on agricultural land could be encouraged through publicly funded agri-environmental schemes as has been seen in the USA (Pourhashem *et al*., 2019). However, waste management regulations currently restrict the application of biochar to land to one tonne per hectare (Environment Agency, 2019). Biochar proponents regard this as a regulatory barrier to widescale deployment prompting efforts to develop a national biochar standard which could enable larger quantities of standard compliant biochar to be applied without the need for a permit from the Environment Agency. Beyond the domain of state governance, market-based actors have begun to express interest in biochar as a source of carbon credits that can be purchased by individuals and firms to offset their carbon emissions. Supporters argue this is vital to the scaling up of the biochar industry. The trading of any form of carbon is highly contested and seen as a form of mitigation deterrence (Carton *et al*., 2020; Markusson, 2022).

Given the widespread uncertainties, we were surprised by the mostly positive tone adopted in the British print news media. We therefore set out to uncover how this discourse was rhetorically structured and what implications for public debate it may have.

## Analytical perspectives

We study biochar benefit reporting as a type of ‘promissory’ or future-oriented discourse. Science and Technology Studies has studied promissory discourses extensively with regard to, among other things, emerging genetic technologies in the field of the sociology of expectations. According to one of the field’s main proponents, Nik Brown: ‘expectations mobilize the future into the present’ (Brown, 2003, p. 3). The future becomes a space onto which visions of technological benefits (most often) are projected. As pointed out in an article on cellular agriculture which is theoretically rooted in the sociology of expectations:

Even before an innovation has emerged and becomes embedded within socio-technical regimes these visions have ‘real’ implications, legitimizing certain trajectories over others, and directing research resources and focus. The core contention is that promissory narratives, future expectations and visions need to be taken seriously because they perform important political and material tasks in the present. (Helliwell and Burton, 2021, p. 181)

Many actors in the biochar field, from scientists, to activists to entrepreneurs, appear to be engaged in such mobilisation by promising that biochar will have multiple benefits in the future, for the global climate as well as for the local allotment. As Brown has pointed out:

Expectations can be performative also in the sense that promises are performative. The phrase ‘I promise X’ is not just a description, it makes the person who enunciates the phrase accountable for doing X (or a version of X). […] this is how early promises and early warnings lead to reactions and sometimes to escalating arguments for and against […]. (2003, p. 3)

Future-making using positive expectations is performative and has normative implications about what should or shouldn’t be done to, for example, make soils better or to mitigate the effects of climate change.

Thus, we are particularly interested in *how* the promises of biochar are made and how positive expectations and normative implications are created. Scholarship has shown that metaphors and other rhetorical devices can be used by experts and the media to shape visions of the past and/or the future to try and affect our social and political actions in the present. They can also be used to orient users (whether as institutions, groups or individuals) to particular possibilities for action and have an effect on material investment (Brown, 2003; Brown and Michael, 2003). ‘The work of metaphor,’ argues the metaphor analyst James Bono, ‘is not so much to represent features of the world, as to invite us to act upon the world as if it were configured in a specific way like that of some already known entity or process’ (Bono, 2001, p. 227). Like promises, metaphors too have a performative force. In respect of GGR, Castree (2020, p. 9) argues that ‘close, critical attention to metaphor could trigger rich debates about key issues’ relating to these emerging technologies.

Here we go beyond the analysis of metaphor as an expectation-creating device to also study a number of other, less visible and subtler lexical and rhetorical devices including hyperbole. ‘Hyperbole’ is ‘a way of speaking or writing that makes someone or something sound much bigger, better, smaller, worse, more unusual, etc., than they are’ (*Oxford Learner’s Dictionary*, online). Hyperbole is a literary/rhetorical device that relies on exaggeration and should not be conflated with hype which is associated with excitement and publicity in relation to institutional promoters of technology. Hype, and patterns of hype and disappointment, have been studied extensively in the sociology of expectations literature; more specific rhetorical devices like hyberbole, less so. Here we study a number of rhetorical devices that are used to attract public attention to an issue and can skew expectations around it, yet might be overlooked in the search for grander narratives of hype. In doing so, we extend an under-developed strand of research in the sociology of expectations by offering a detailed linguistic analysis at the microlevel of the text.

## Sampling and method

**Figure 3.**
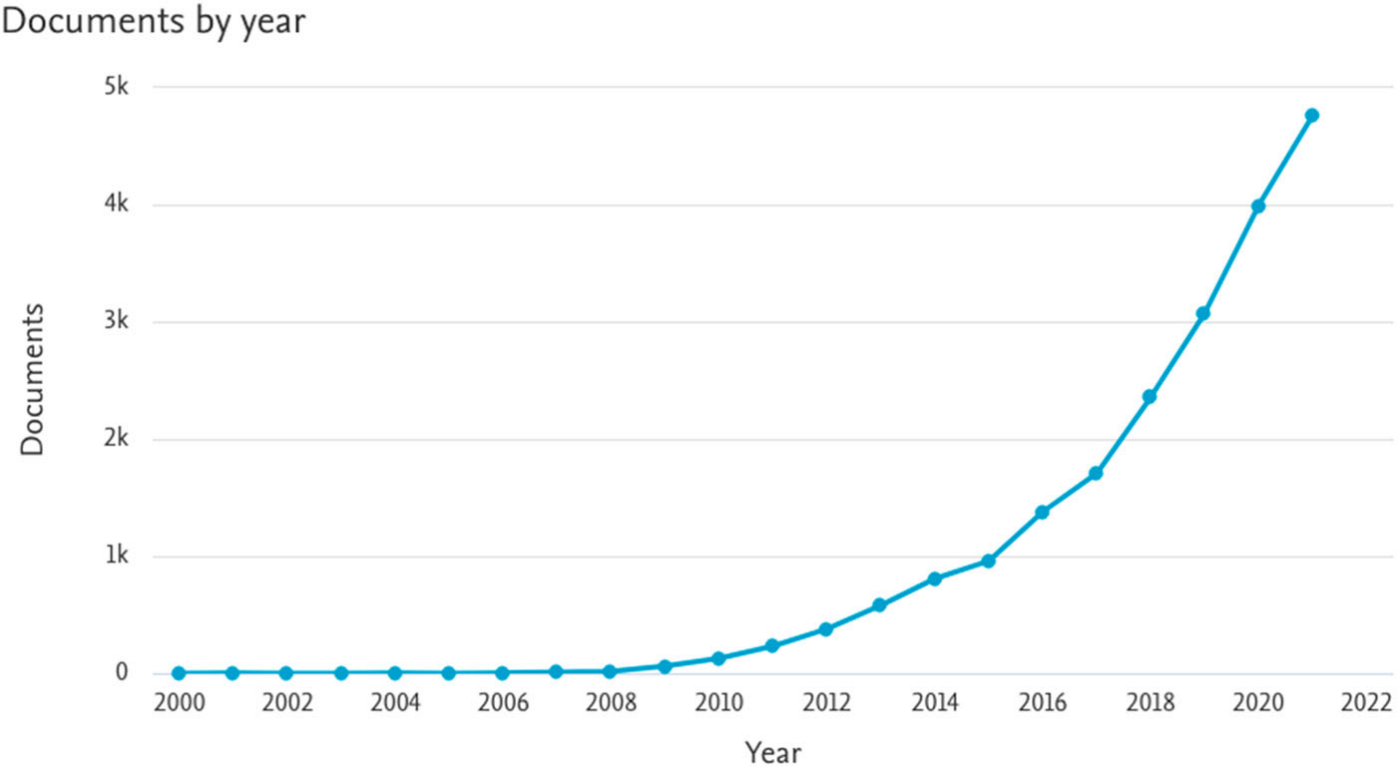
Scientific articles on biochar published since 2000.

Biochar has been discussed by climate, soil, agricultural and other scientists since around the year 2000, when the first scientific article appeared under the title *Biochar: From the straw-stalk of rapeseed plant* (Karaosmanoǧlu *et al*., 2000). A decade later, scientific output began to gradually increase, as one can see on Figure 3, extracted from *Scopus*, a database of research articles.

**Figure 4.**
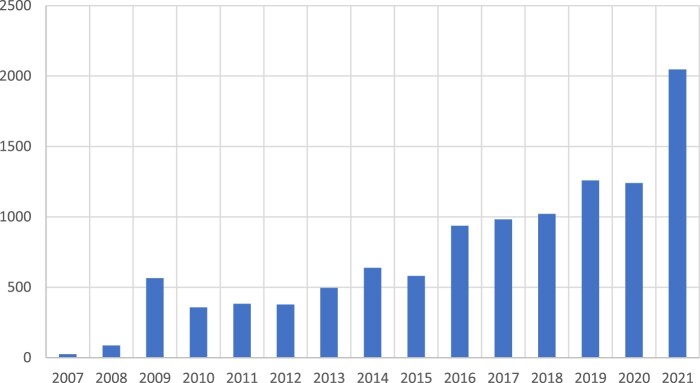
‘Biochar’ in All English Language News (Nexis), 2007–2021.

To find out how news outlets covered the emerging topic of biochar, we searched the news database Lexis Nexis on 4 March 2022 using the search term ‘biochar’ on a high similarity setting which excludes most duplicates. We found that in the Lexis Nexis category All English Language News, 10,993 articles have been published on biochar since 2007. The distribution of that coverage did not follow the curve of scientific output exactly, but coverage increased in 2009, and somewhat after 2015 and 2018 and in 2021 (see Figure 4). It should be said, however, that the number of scientific articles is higher than the number of English news articles, which might account for a smoother curve.

Of the 10,993 items in All English Language News, 408 articles were published in British national newspapers, the focus for our analysis, with the first article being published in 2008. We homed in on those newspapers listed as the top four publishing on biochar, all broadsheets but of different political orientations, with *The Guardian* on the left, *The Daily Telegraph* on the right, with *The Independent* leaning towards the left and *The Financial Times* being seen as centre-right. Together, these national broadsheets published a total of 100 articles, a number suitable for in-depth qualitative analysis: *The Guardian* (*N* = 54 articles; first article 2008), *The Daily Telegraph* (*N* = 19 articles; first article 2009), *The Independent* (*N* = 14 articles; first article 2009) and *The Financial Times* (*N* = 13 articles; first article 2008). Although tabloid newspapers have a wider readership, they have published only a handful of articles on biochar so were excluded from the analysis. Most articles appeared towards the beginning of the period under investigation, with interest tapering off towards the end of our research period. The first article appeared in 2008 with no articles appearing in 2022 (until 4 March 2022 when the dataset was extracted). The peak of reporting occurred in 2009 when 26 articles appeared in UK broadsheet newspapers. Two smaller peaks occurred in 2015 (12 articles) and 2021 (10 articles).

We employed a qualitative thematic analysis, which is ‘a method for identifying, analysing and reporting patterns (themes) within data’ (Braun and Clarke, 2006, p. 78). Following the analytic steps outlined in Jaspal (2020), both the headline and the main body of each article were subjected to thematic analysis. We read and re-read the articles to familiarise ourselves with the broader themes that we subsequently discussed analytically.

We made initial observations that captured the essential qualities of each article, the units of meaning and dominant rhetorical techniques. After that, we paid special attention to how certain aspects of biochar were made rhetorically salient, in the sense of ‘making a piece of information more noticeable, meaningful, or memorable to audiences’ (Entman, 1993, p. 53). We paid close attention to the use of evaluative adjectives and verbs (words which are used to appraise subjects or objects), the use of metaphors, repetition and the association of words or phrases with ‘culturally familiar symbols’ (Entman, 1993).

Adjectives and verbs are words that are easily overlooked but can affect the way a new phenomenon is positioned, positively or negatively. As Millar *et al*. found in relation to promotional discourse in grant applications, adjectives are important ‘because they are the word class most associated with evaluation’ (Millar *et al*., 2022). The importance of small words like adjectives emerged from our thematic analysis. Unlike Millar *et al*., however, we did not carry out a formal frequency analysis of their use. In his *Glossary of English Grammar*, Geoffrey Leech writes that ‘[a]djectives are a large class of words (for example, good, bad, new, accurate, careful) which define more precisely the reference of a noun or pronoun’ (Leech, 2006, p. 6). We looked at adjectives like ‘natural,’ for example, in various positions, as in ‘natural biochar,’ ‘biochar is natural,’ or ‘biochar is a natural solution.’ Adjectives deserve our attention as they define and evaluate a new phenomenon in certain ways, and thus position it linguistically, politically and ideologically. We also examined verbs which turned out to be central to the metaphorical framing of biochar.

Metaphors allow the understanding of new, unfamiliar, and abstract phenomena in terms of more concrete subject matters. They foster understanding of complex issues by referring to concepts and objects from everyday experience. In their seminal book on *Metaphors We Live By*, Lakoff and Johnson (1980) distinguished between conceptual metaphors and the linguistic expressions that can be derived from them. For example, life is a Journey is a conceptual metaphor (conventionally represented in small capitals) which maps aspects of journeys onto how we conceptualise life. ‘We have reached the end of the road’ or ‘my life is at a cross roads’ are verbal expressions based on or subsumed under this conceptual metaphor. This approach to metaphor analysis has been successfully applied to the study of scientific and news texts. As Nelson *et al*. (2015) have made clear,

[m]etaphor analysis involves identifying metaphorical language and then articulating the underlying metaphorical concepts […]. For example, phrases such as ‘the genome is read’ and the ‘first draft of the human genome’ can be grouped into the underlying metaphorical concept ‘the genome is text.’ (pp. 60–61)

Metaphors can be found, paradoxically, by reading a text literally. For example, in ‘they read the human genome,’ we notice a clash between something one does to a text and something deeply biological and ask ourselves ‘do we literally read the genome?,’ before answering ‘no this must be a metaphor.’ This is the essence of the more elaborate and well-stablished ‘metaphor identification procedure’ (see Pragglejaz Group, 2007). We also paid attention to rhetorically salient repetitions or lists of words or phrases and allusions to culturally salient symbols, even if they were not strictly metaphorical.

We discussed our respective initial codes, which included general tone, particular forms of language and emerging patterns in the data. These codes were collated into preliminary themes, lexical tools and rhetorical techniques that were subsequently arranged into a coherent structure that reflected the overall thematic analysis. We refer to these devices as ‘promissory’ in the text because they are crucial to shaping expectations around biochar.

We now provide some additional context before moving on to examine the lexical and rhetorical tools used to make one major theme – the benefits of biochar – prominent. We provide extracts from the articles that exemplify how they were used to create a promissory discourse and associated expectations around biochar.

## Empirical analysis

Biochar has not been discussed extensively in the print news media in the UK. Sometimes a whole year or several years go by without a mention. Biochar was first reported in *The Guardian* on 27 November 2008 in an article promoting a book by Chris Goodall called ‘10 technologies to save the planet.’ This was also the year that the company ‘Carbon Gold’ was founded by Craig Sams and David Morrell. Sams has been one of the key-players in making biochar public in the news and selling it to gardeners and allotment users.

A number of key actors and events drove early reporting on biochar. For example, the United Nations Climate Change Conference in 2009, commonly known as the Copenhagen Summit, was held in Copenhagen, Denmark. In the same year an article on biochar appeared in the journal *Nature* entitled ‘The bright prospect of biochar’ (Kleiner, 2009), and the UK Biochar Research Centre was founded at Edinburgh University, which is still active today. Interest in biochar increased after the 2015 Paris Agreement and after 2018, when biochar was ‘included as a promising negative emissions technology’ (NET) in the Special Report on Global Warming of 1.5°C (IPCC, 2018, Chapters 2 and 4) produced by the Intergovernmental Panel on Climate Change (IPCC) (Hansson *et al*., 2021). Since then, biochar has been discussed as part of debates around net zero and interest and investment have been growing, as testified by the public funding of several research projects in the UK in 2021.

We found that, overall, the tone of media coverage was positive; in early reporting even enthusiastic. The discursive focus of biochar reporting seems to have been that it promises multiple benefits now and in the future. This mirrors the tone and trend in scientific review articles, which also focused on benefits (see Latawiec *et al*., 2016). Some negative aspects of biochar were discussed as well, most importantly through the lens of parody, as we will see later. In the following, we shall explore how the benefits of biochar were rhetorically represented in the promissory narratives told by various key actors in British broadsheet newspapers and how some of these narratives were rhetorically contested.

The reported future benefits of biochar range from offering solutions to dealing with climate change (the focus of left-leaning papers) to being a boon to gardeners (the focus of right-leaning papers). Such benefits were discussed by scientists, both popular and otherwise; celebrities endorsing market-based solutions to greenhouse gas removal, such as entrepreneur Richard Branson and fashion designer Vivienne Westwood; eco business leaders, such as Craig Sams of Carbon Gold as well as other, lesser-known entrepreneurs and representatives of climate tech companies.

The gardening benefits were the focus of reporting in *The Daily Telegraph*, while the environmental benefits were discussed mainly in *The Guardian*. The promotion of biochar as a boon for gardeners, in particular regarding boosting soil fertility and being able to replace peat, came mainly from Sams, some gardening journalists and horticulturalists, of which some are associated with the Royal Horticultural Society. A minor, counter theme was identified – of biochar being associated with risks or harms, especially land-use and other environmental threats, similar to those related to biofuels. This was mainly driven by the environmentalist George Monbiot writing for *The Guardian*, as well as some environmental NGOs including Biofuel Watch and Friends of the Earth. When writing about the risks of biochar, Monbiot challenged a number of biochar enthusiasts such as James Lovelock, Jim Hanson, the author Chris Goodall and the climate campaigner Tim Flannery.

The benefits of biochar were postulated mainly with relation to climate change mitigation and soil improvement, which in turn also related to issues around gardening and – to a much lesser extent – farming. We found that these claimed-for benefits of biochar were made salient through the use of a variety of rhetorical devices that all boosted and in some instances exaggerated such benefits. This mostly happened through the use of distinctive adjectives and verbs; the use of a crucial metaphor; the use of two-, three-, or four-part lists; the use of hyperbole; and through allusions to literary and cultural symbols that give biochar a certain mystique. Risks and uncertainties were only a minor concern in reporting. These were most saliently positioned in opposition to the benefits discourse (again picking up issues of climate change mitigation, farming and gardening), indeed parodying it in places. Regulation and governance issues were thematically almost absent and not made rhetorically salient in any noticeable way.

### Promissory adjectives

Many articles (and the quotes within them by key proponents of biochar) contained a series of positively connotated adjectives. These lexical choices promise biochar to be a beneficial, indeed, simple, cheap and even ‘astonishing’ technology. As far as we could see, no negatively connotated adjectives were used in our corpus. This difference is indicative of a certain enthusiastic style of discourse that prevailed in the media coverage, especially in early reporting.

A first group focused on the fact that biochar was supposedly ‘low-tech,’ ‘simple,’ ‘relatively cheap’ and ‘inexpensive’ and ‘suitable for gardeners,’ allotment users and smallholders, as well as farmers, particularly those in the global south. As one article said: ‘Make charcoal and bury it in the ground: a simple and effective form of carbon capture and storage’ (*The Guardian*, 2009a).

In the first article in our corpus, Chris Goodall claimed that biochar was an ‘outstanding’ cheap and simple (low tech) climate solution that sequesters carbon ‘effectively for ever’ (Goodall, 2008). Here we can see the use of some core adjectives, such as ‘cheap and simple,’ but also more hyperbolic ones like ‘outstanding,’ to which we will return later. One other adjective that appeared in the context of promising cheapness and simplicity used by Goodall was ‘astonishing.’

The ‘astonishing’ low-tech nature, simplicity and cheapness of biochar was often positioned in opposition to the high-tech, complex and expensive nature of carbon capture and storage. This argumentative move of opposing low-tech, also sometimes called natural, and high-tech, or artificial, solutions contributes to turning biochar into a more promissory and achievable technology. Even though some ‘natural’ approaches to climate change mitigation are equally technical and risky, those framed as natural are generally seen as ‘more beneficial, cost effective, mature, and democratic than ostensibly artificial counterparts’ (Osaka *et al*., 2021, online). The question is: ‘Would lay publics still prefer natural solutions to carbon removal, if they are informed about the inevitable technological framing and shaping of them?’ (Markusson, 2022). That is a question for future research.

*The Independent* positioned biochar as an ‘attractive’ approach to storing carbon in soil idea and even likened it to geoengineering: ‘The capture of sustainably produced plant carbon as biochar is perhaps the most attractive approach to soil geoengineering’ (*The Independent*, 2009a). Later that year, Sams said in a leader column that biochar was an ‘exciting and realistic means of reducing greenhouse gas levels’ (Sams, 2009). Biochar was framed as both ‘exciting’/‘attractive’ and ‘realistic,’ again framing the promise as achievable – as opposed to what are implied to be complicated and difficult technologies.

Another group of adjectives focused on what biochar promised to be in the future once deployed, namely ‘eco-friendly,’ ‘effective,’ ‘long-lasting’ and ‘stable.’ In fact, it was portrayed as ‘benign,’ and, above all, of ‘ancient’ origin, through reference to Amazonian *terra preta* or dark earth (Leach *et al*., 2012; Soentgen *et al*., 2017), giving it a patina of what one may call good old fashioned and traditional ways of working.

In an article using wordplay in the title, ‘Black is the new green’ an allusion is made to Craig Sam’s previous entrepreneurial venture, the chocolate brand Green & Black’s. Another article in *The Financial Times* used a string of future-oriented compound adjectives in its title: ‘The long-lasting, eco-friendly, carbon-storing wonder stuff’ (Maccoby Berglof, 2011) – we shall come back to ‘wonder stuff’ when talking about biochar’s mystique. All this makes biochar both attractive and valuable. Let us now turn to verbs.

### Promissory verbs and crucial metaphors

Verbs were employed first of all to describe what biochar actually does. We find verbs like ‘bury,’ ‘lock up,’ ‘lock away,’ ‘soak up’ and ‘suck up,’ ‘capture’ and ‘sequester,’ as well as ‘fix’ or ‘stabilise.’ All refer to biochar’s supposed ability to ‘absorb’ and ‘retain’ carbon dioxide and thus mitigate climate change. Other words were used to describe what it does once buried in the soil, such as ‘trap’ (nutrients), ‘fertilise,’ ‘regenerate,’ ‘transform’ (waste) and ‘boost’ fertility. Sometimes alliteration is used to contrast the beneficial aspects of biochar to the use of fossil fuels, as in ‘bury not burn.’

We found that two conceptual metaphors structured the use of several of these verbs in our corpus. On the one hand, biochar was conceptualised as a sponge (‘soaking,’ ‘sucking’), on the other as a secure containment mechanism that captures and locks up harmful elements (‘trapping,’ ‘catching,’ ‘burying,’ ‘storing,’ ‘locking up’). We will refer to these two (conceptual) metaphors as biochar is a sponge and biochar is containment. Both metaphors map something familiar, a sponge or, say, putting something into a secure container, onto something unfamiliar and thus make it accessible to people and also more desirable. They promise that the bad stuff that pollutes our atmosphere, carbon, can be easily removed, securely stored away, forgotten and even turned into something good, for the soil. These metaphors have a performative and normative force. They make us think that biochar *should* be used to clean up the soil, garden and planet by removing harmful substances or polluters. As Sally Wyatt said in 2004: ‘metaphors do not simply have a descriptive function but […] they also carry normative connotations’ (p. 157).

Interestingly, in some recent reporting, the promises of biochar seem to be on the verge of becoming reality: ‘*should*’ becomes ‘*can*.’ *The Guardian* reported on the first biochar trials under the headline ‘Trials to suck carbon dioxide from the air start across the UK’ (Carrington, 2021). The *Financial Times* reported on a new Finnish company called *Carbo Culture* and pointed out that ‘Its pilot reactor, which turns biomass into biochar storing carbon in a stable form for a thousand years, has just begun operation in California and the company is planning to open its first commercial plant in Helsinki in 2024 selling renewable heat’ (Thornhill, 2021).

All these promissory properties of biochar are highlighted through the lexical choice of certain verbs and related metaphors. Biochar promises to ‘alleviate,’ ‘help,’ ‘enhance,’ ‘improve,’ ‘bolster,’ ‘promote’ and ‘increase,’ as well as ‘prevent,’ ‘reduce,’ ‘restore,’ ‘retain,’ ‘reverse’ and even ‘revolutionise’ all sorts of things above the earth, in the atmosphere, and below the earth where plants grow. For example, a *Guardian* article from 27 August (*The Guardian*, 2009b) said: ‘it could bolster global attempts to address climate change through cuts in greenhouse gas emissions.’ It is therefore not surprising that biochar has been praised as a ‘technology of repair’ (Leach *et al*., 2012, p. 286).

A good example of the use of promissory verbs, that is, words which point to future positive impacts, such as ‘improve’ and ‘prevent,’ can be found in an article in *The Independent* – but this way of organising ideas around biochar is quite pervasive in all the newspapers we analysed:

So what is biochar? Basically, it’s the name given to charcoal that is used to improve soil. It is said to improve water retention, promote the take-up of nutrients and increase productivity. Best of all, the process by which it is produced – called pyrolysis – eliminates as far as possible the presence of oxygen. This prevents combustion, so unlike burning plant matter or agricultural waste, or letting it decompose, it does not release huge amounts of carbon dioxide into the atmosphere. (Summerley, 2012)

A similar suite of verbs is used in a *Guardian* article saying that biochar ‘can significantly boost crop productivity, reduce nitrous oxide and methane emissions and improve soil structures’ (*The Guardian*, 2009c). However, biochar is not only made attractive through adjectives and verbs; the promissory discourse around biochar goes further and to understand it, we need to go back to some well-established rhetorical strategies used in persuasive discourse.

### Promissory oratory

In classical rhetoric, a bicolon, tricolon, or tetracolon is a sentence pattern with two, three, or four clearly defined parts, usually independent clauses and of increasing power. It has been used in political discourse since ancient times and more recently in advertising (Zimmer, n.d.). Famous examples are ‘Veni, vidi, vici,’ or ‘blood, sweat and tears.’ Political speeches make ample use of more extended forms of this type of repetition. Former British prime minister Tony Blair once said for example ‘It means exposing as the rubbish it is, the propaganda about America and its allies wanting to punish Muslims or eradicate Islam. It means championing our values of freedom, tolerance and respect for others. It means explaining why the suppression of women and the disdain for democracy are wrong’ (BBC, 2005). Here the tricolon is combined with the use of anaphora and parallelism, that is, the use of similar structures in a pair or string of phrases or clauses (see Freeman, 2015).

According to Max Atkinson, a specialist in political rhetoric, rhetorical devices, such as three-part lists and contrasts, are consistently effective in eliciting audience applause for political speeches (Atkinson, 1984). In our case, these and similar constructions invite applause for biochar – using many of the words we have discussed above. Such words and lists are a central feature of the promissory discourse promoted by some scientists, entrepreneurs and some of the journalists that quote them.

We often find promissory eulogies like this: ‘Food crops grow better. Trees planted in biochar often have better root systems. Crop yields are improved’ (Goodall, 2009). Or this:

Morrell, who founded Future Forests, which later became the Carbon Neutral Company, said: ‘Biochar is the only technology that enables us to take invisible carbon dioxide out of the atmosphere, transform it into black lumps of pure carbon and, by ploughing it into the soil, prevent it from going back into the atmosphere.’ (*The Guardian*, 2009b)

Such three- or four-part lists carry through to articles and quotes a decade later, when Jessica Murray wrote in *The Guardian*: ‘It traps carbon in the ground for centuries, boosts plant growth, provides a sustainable heat source and could even reduce methane emissions from cows’ (Murray, 2019). This pattern of repetitive praise also appears in the *Daily Telegraph*; ‘It may provide a carbon sink, it may save degraded soils, it may alleviate rural poverty’ (Leendertz, 2013); and when summarising a radio programme on the matter, an article pointed out: ‘biochar, a high-carbon form of charcoal that can improve soil fertility, reduce pollutants, and help to strengthen the Earth against the ravages of flooding and drought’ (*The Daily Telegraph*, 2021).

### Promissory exaggeration

Expectations about all the good things that biochar can do are not only strengthened by verbs like ‘boost’ and tricolons not dissimilar to ‘veni, vidi, vici,’ but also by the use of adjectives and nouns like ‘outstanding,’ ‘admirable,’ ‘extremely promising,’ as well as ‘potential,’ ‘promise,’ ‘miracle’ and ‘wonder.’ The last two words in particular do not describe demonstrable properties of biochar, but, we suggest, exaggerate or overstate its potential, and thus engage in hyperbole.

Tim Miller, a project manager of European Bioenergy at Aston University wrote in *The Guardian*: ‘In fact, its by-product – biochar – can be used to increase crop yields. And by using heat instead of incineration, it produces no emissions. In short, there are no downsides’ (Miller, 2012). To say of something that it has ‘no downsides’ is a grand statement! Simon Shackley, a social science lecturer at the University of Edinburgh and well-known biochar expert, goes one further and says: ‘It’s wonderful stuff’ (Harvey, 2009).

There are also instances where the virtues of biochar are extolled intertextually by referencing for example the famous speech by Martin Luther King ‘I have a dream.’ *The Guardian* quotes a fan of biochar, Laurens Rademaker, as saying: ‘I have a dream that one day the driver of an electric Hummer [powered with energy produced by making biochar] will be our biggest climate hero’ (*The Guardian*, 2009d). It is indeed a ‘super charcoal’ that locks up carbon (Walker, 2012). Here biochar is personified as a hero of climate mitigation, even a ‘super-hero.’

Reporting on an article published in *Nature*, *The Guardian* used a well-worn cliché or metaphor in its reporting that is more often used in health reporting, the ‘silver bullet’ which positions an invention or intervention as simple and almost magical: ‘The journal *Nature* Reports Climate Change said that biochar “could be the closest contender yet for a silver-bullet solution to climate change”’ (*The Guardian*, 2009b). Creating positive expectations about biochar is not only what some scientists do, it is what entrepreneurs do as well, even more so. This is why Sams called his product ‘Carbon Gold,’ for example and another company calls itself ‘CoolTerra.’ By using the word ‘gold,’ Sams associates his product with something that is priceless, holds value and stands the test of time. Using wordplay, *The Guardian* entitled one of its articles ‘Turning charcoal into carbon gold’ (2009b) – alluding to alchemy. This brings us to biochar’s ‘mystique.’

### Promissory mystique

In addition to making biochar benefits salient through word choice, argumentative repetition and hyperbole, we found one more rhetorical move that characterises biochar’s promissory discourse. Some articles used creative allusions to well-known literary texts to convey a mystical status to biochar. The media analyst Entman would have said they linked biochar to ‘culturally familiar symbols’ (1993, p. 53).

Two newspaper articles refer to Philip Pullman’s books *His Dark Materials* (a series of fantasy novels published between 1995 and 2000) in their headlines. On 27 September 2009 *The Independent* reported on an interview with Sams under the headline: ‘His dark materials: The man behind Green & Black’s chocolate wants to save the planet with charcoal’ (*The Independent*, 2009b). Ten years later *The Guardian* combined the dark materials allusion with an alchemy metaphor in this headline: ‘This dark material: the black alchemy that can arrest carbon emissions’ (Murray, 2019).

When discussing the risks and benefits of emerging technologies, famous book titles, such as *Frankenstein,* have been used to evoke fears of biotechnologies. In the case of ‘dark materials’ the use of a book title does not refer to any content of story line though, but just trades on the darkness of the material that is charcoal, giving biochar a sort of mystique. Nelkin and Lindee (2004) used the word ‘mystique’ in their analysis of the DNA double helix which once gave biotechnology a sense of mystique. Here allusions to book titles are used to praise biochar, not to induce fear, as in the case of ‘Frankenstein.’ Indeed, the reference to alchemy opens up a magical promissory space where one can dream about the transmutation of matter, in particular with attempts to convert base metals into gold or finding the elixir of life. In this case it is a dream of turning waste matter into carbon storage and soil fertilisation. As we have seen above, one headline called biochar ‘long-lasting, eco-friendly, carbon-storing wonder stuff’ (Maccoby Berglof, 2011).

This mystical and wondrous quality of biochar, this ‘dark material,’ this ‘dark alchemy,’ was also promoted through telling the story of *terra preta*, the ancient, anthropogenic dark soil found in the Amazon. The environmental reporter Fiona Harvey wrote: ‘Terra preta, modern analysis has proved, is one of the last remaining traces of pre-Columbian agriculture in the Amazon basin … the key ingredient in terra preta, and what gives it its dark colour, is charcoal’ (Harvey, 2009). In *The Daily Telegraph*’s gardening section, which featured biochar quite prominently, one can read: ‘Biochar is “the oldest new thing you’ve never heard of”, to quote a phrase coined by Wae Nelson, a US biochar expert’ (*The Daily Telegraph*, 2013). Here we can see a rhetorical appeal to tradition at work, also known as *argumentum ad antiquitatem*: the old justifies the new and the benefits of the new – in this case helping to reduce global warming. In an article entitled ‘Cool planet’ referencing a business called CoolTerra, we read for example:

Inspired by the fertility of the biochar-rich Amazonian soil called terra preta, enthusiasm for biochar soil and water additives has grown in recent years. Biochar is said to enhance soil quality by increasing its microbe content – improving yields while requiring less water and fertilizer. In water, it acts like a filtration agent. And biochar is potentially a tool to fight climate change because it can sequester and hold carbon in the soil for a long time. (Sharma-Sindhar, 2014)

Biochar is, it seems, a jack of all trades. It is not, as the reporter stresses, just ‘a fantasy soil.’

### Parodying promises and dashing hopes

To challenge these expectations and promissory discourses based on hyperbole and mystique, some commentators have used irony, parody or sarcasm.

The writer and environmentalist George Monbiot employed these techniques to good effect at the beginning of the biochar infatuation, when he wrote:

According to the magical thinkers who promote it, the new miracle stops climate breakdown, replaces gas and petroleum, improves the fertility of the soil, reduces deforestation, cuts labour, creates employment, prevents respiratory disease and ensures that when you drop your toast it always lands butter side up. (I invented the last one, but give them time). (Monbiot, 2009)

He sees biochar as magical thinking and a crazy delusion – using what one may call satirical hyperbole! The reason for this scepticism is that he thinks biochar is unlikely to scale up and that it is beset by the same problems as biofuels. This is echoed by Mike Childs, climate campaigner with Friends of the Earth, who said, in a slightly more conciliatory tone: ‘The problems with biochar are largely the same as biofuel. If you manage it properly then making limited amounts is OK, sensible and useful’ (*The Guardian*, 2009b). Proponents compared biochar with carbon capture and storage, even geoengineering, but argue it is cheaper and simpler. Opponents compare it with biofuels and stress that it will have the same problems with regard to land use, large-scale deforestation, soil erosion and so on. As one *Guardian* reader said ‘Biochar can be a wonderful component to a climate change mitigation program. However, it does not make sense, as some would suggest, to grow, kill and then char trees’ (Rice-Oxley, 2019) – using a triplet of verbs that turn the positive into a negative framing.

The best parody of the ‘wonder’ or ‘miracle’ framing of biochar can be found in the same Monbiot article:

Whenever you hear the word miracle, you know there’s trouble just around the corner. But no matter [how] many times they lead to disappointment or disaster, the newspapers never tire of promoting miracle cures, miracle crops, miracle fuels and miracle financial instruments. […] So welcome ladies and gentlemen to the new miracle. It’s a low-carbon regime for the planet that makes the Atkins diet look healthy: woodchips with everything. (Monbiot, 2009)

This parody of the biochar ‘miracle’ and the sarcastic tone of Monbiot’s article brings this promissory discourse down to earth and makes it amenable to understanding and to criticising. Talking about a ‘low carbon diet’ brings the reduction of greenhouse gases down to the human scale (see Nerlich *et al*., 2011). We might not know how to engage in climate change mitigation but we all know about diets. However, Monbiot doesn’t use this phrase to exhort people to reduce their carbon footprint. Instead, Monbiot turns the phrase on its head by talking about the Atkins diet where people eat mainly meat, and he adds chips (or as Americans would say ‘fries’) to that ‘diet,’ making clear that using biochar as a climate ‘dieting’ device is not a good thing.

## Conclusion

As public awareness of biochar is still low, print news media can set expectations and shape public debate around this emerging technology for greenhouse gas removal (GGR). Adopting the theoretical lens of the sociology of expectations and using a detailed rhetorical analysis, this article has studied the way UK broadsheet newspapers report on biochar and draw attention to some aspects at the expense of others.

The overarching research question was: How is biochar represented in the UK print news media, specifically the broadsheet newspapers? In particular, what major themes and topics were explored and who promoted them? The second, more specific, question was: Which rhetorical tools were used to promote biochar and to contest such promotion? That is, how was biochar made rhetorically salient for readers?

One major theme or topic dominated the newspapers, namely that biochar promises many benefits in the future, from offering solutions to dealing with climate change to being a boon to gardeners. Alongside this major theme ran a minor theme or counter theme of biochar being associated with risks or harms, especially land-use and other environmental threats, similar to those related to biofuels. Left-leaning newspapers enthused most about the potential benefits of biochar for climate change mitigation (or, indeed, critiquing such enthusiasm) and Right-leaning ones enthused most about the potential benefits for gardening; the former having a collectivist focus, the latter an individualist and also entrepreneurial one.

To study how these themes were rhetorically presented, we focused in particular on linguistic or rhetorical strategies and their use in constructing promissory narratives of biochar as a technology with the potential to contribute to GGR and soil health. This type of analysis can provide initial insights into societal understanding of biochar and how newspapers and key actors they interact with are creating a horizon of expectations about the contribution of this relatively unknown technology to addressing the climate crisis.

The analysis found a spectrum of rhetorical tools being used, ranging from the use of evaluative adjectives and metaphorical verbs to the repetition of phrases and finally the allusion to cultural symbols that confer a certain mystique to biochar. These rhetorical devices were employed mostly by scientists, activists and entrepreneurs to position biochar as something ‘beneficial,’ as something the use of which should be aspired to in order to improve the soil, the garden and save the planet. There were only very rare voices of criticism, using, for example, the rhetorical tool of satire or parody to counter such a framing and its performative and normative implications, namely that it should be adopted.

Amongst the rhetorical devices studied, hyperbole and cultural allusions which surrounded biochar with a promissory mystique were used to overstate the benefits of biochar. In contrast, parody and satire were used to undermine those engaged in emphasising the benefits of biochar. These rhetorical manoeuvres fill a public awareness gap surrounding biochar in a very particular way, positioning biochar overwhelmingly as a moral object that ought to be supported by the public. This sets the backdrop to public acceptance of biochar as a new carbon removal and garden fertilisation technology, rather than opening up a space for public discussion and deliberation.

Biochar is thus largely framed as a ‘technology of repair’ (Leach *et al*., 2012, p. 286) based on its ability to soak up and store carbon, improve soil health, increase crops yields, and reduce pollutants. Such promissory framing can render the uncertainties or unintended consequences associated with biochar invisible. When speculations are articulated about biochar, voices emerge that highlight some of the difficulties of upscaling this technology and other risks attached to it, especially environmental ones. Public perceptions and expectations are therefore pulled in two, unequal and opposing directions regarding biochar, between believing the many promises made or listening to the few voices of caution.

This article is among the first studies in the STS literature to examine biochar in print news media and to carry out a detailed microlevel examination of a wide range of rhetorical devices, including specific word choices, that can be used in promissory discourses of an emerging technology. This offers a new perspective for the sociology of expectations interested in analysing the more imperceptible steering of societal discourses, over and above grander narratives of hype, in the print news media in particular. Studying promissory discourse at the microlevel within texts could broaden the methodological scope of the sociology of expectation and could be usefully applied to other climate control technologies.

This research does not tell us how audiences respond to the representation of biochar in the print news and other media. Further research would be needed to gain insights into audience views, understandings, and beliefs surrounding biochar and thereby explore the various effects of the rhetorical strategies identified here. A reception analysis could be conducted, either using interviews, focus groups, or analysis of below the line comments (Price, 2021). This research has only focused on the lexical and rhetorical tools used to make the benefits of biochar prominent in news coverage and to create a promissory discourse around biochar. A wider analysis would be necessary to investigate the more critical rhetorical strategies associated with biochar, both within and beyond the print news media.

## References

[CIT0001] Anderson, A., Stuart, A., Alan, P. and Wilkinson, C. (2005) The framing of nanotechnologies in the British newspaper press, *Science Communication*, 27(2), pp. 200–220.

[CIT0002] Atkinson, M. (1984) *Our Masters’ Voices: The Language and Body Language of Politics* (London: Routledge).

[CIT0003] BBC (2005) Full text: Blair speech on terror, *BBC*, July 16. Available at http://news.bbc.co.uk/1/hi/uk/4689363.stm (accessed 16 June 2022).

[CIT0004] Bellamy, R., Chilvers, J. and Vaughan, N. E. (2016) Deliberative mapping of options for tackling climate change: Citizens and specialists “open up” appraisal of geoengineering, *Public Understanding of Science*, 25(3), pp. 269–286. doi:10.1177/0963662514548628 25224904 PMC4819797

[CIT1003] Bellamy, R., Lezaun, J. and Palmer, J. (2017) Public perceptions of geoengineering research governance: An experimental deliberative approach, *Global Environmental Change*, 45, pp. 194–202. doi:10.1016/j.gloenvcha.2017.06.004

[CIT0005] Bezerra, J., Turnhout, E., Vasquez, I. M., Rittl, T. F., Arts, B. and Kuyper, T. W. (2019) The promises of the Amazonian soil: shifts in discourses of Terra Preta and biochar, *Journal of Environmental Policy and Planning*, 21(5), pp. 623–635. doi:10.1080/1523908X.2016.1269644

[CIT0006] Braun, V. and Clarke, V. (2006) Using thematic analysis in psychology, *Qualitative Research in Psychology*, 3(2), pp. 77–101.

[CIT0007] Bono, J. J. (2001) Why metaphor? Toward a metaphorics of scientific practice, in: S. Maasen and M. Winterhager (Eds) *Science Studies: Probing the Dynamics of Scientific Knowledge*, pp. 215–234 (Bielefeld: Transcript).

[CIT0009] British Society of Soil Science (2021) Science note: Soil carbon. Available at https://soils.org.uk/wp-content/uploads/2022/05/BSSS_Science-Note_Soil-Carbon_Final_May22_75YRS_DIGITAL.pdf (accessed 6 December 2022).

[CIT0010] Brown, N. (2003) Hope against hype-accountability in biopasts, presents and futures, *Science & Technology Studies*, 16(2), pp. 3–21.

[CIT0011] Brown, N. and Michael, M. (2003) A sociology of expectations: Retrospecting prospects and prospecting retrospects, *Technology Analysis & Strategic Management*, 15(1), pp. 3–18.

[CIT0012] Buck, H. J. (2019) *After Geoengineering: Climate Tragedy, Repair, and Restoration* (London: Verso).

[CIT0013] Carbon Gold (2022) Biochar explained. Available at https://www.carbongold.com/how-to-use-biochar/ (accessed 6 December 2022).

[CIT0014] Carrington, D. (2021) Trials to suck carbon dioxide from the air to start across the UK, *The Guardian*, May 25.

[CIT0015] Carton, W., Asiyanbi, A., Beck, S., Buck, H. J. and Lund, J. F. (2020) Negative emissions and the long history of carbon removal, *WIREs Climate Change*, 11(6), pp. 1–25. doi:10.1002/wcc.671

[CIT0016] Castree, N. (2020) The discourse and reality of carbon dioxide removal: Toward the responsible use of metaphors in post-normal times, *Frontiers in Climate*, 2(December), pp. 1–10. doi:10.3389/fclim.2020.614014

[CIT0017] Chilvers, J. and Kearnes, M. (2020) Remaking participation in science and democracy, *Science Technology and Human Values*, 45(3), pp. 347–380. doi:10.1177/0162243919850885

[CIT0018] Clare, A., Barnes, A., McDonagh, J. and Shackley, S. (2014) From rhetoric to reality: Farmer perspectives on the economic potential of biochar in China, *International Journal of Agricultural Sustainability*, 12(4), pp. 440–458. doi:10.1080/14735903.2014.927711

[CIT0019] Committee on Climate Change (2020) Land use: Policies for a net zero UK. Available at https://www.theccc.org.uk/publication/land-use-policies-for-a-net-zero-uk/ (accessed 6 December 2022).

[CIT0020] Entman, R. M. (1993) Framing: Toward clarification of a fractured paradigm, *Journal of Communication*, 43(4), pp. 51–58. doi:10.1111/j.1460-2466.1993.tb01304.x

[CIT0021] Environment Agency (2019) Storing and spreading biochar to benefit land: LRWP61. Available at https://www.gov.uk/government/publications/low-risk-waste-positions-landspreading/storing-and-spreading-biochar-to-benefit-land-lrwp-61 (accessed 6 December 2022).

[CIT0023] Freeman, D. (2015) Tricolon. Available at https://www.vernaculardiscourse.com/tricolon.html (accessed 16 June 2022).

[CIT0024] Goodall, C. (2008) The 10 big energy myths, *The Guardian*, November 27.

[CIT0025] Goodall, C. (2009) Biochar: Much is unknown but this is no reason to rule it out, *The Guardian*, March 24.

[CIT0027] Green Alliance (2019) Cutting the climate impact of land use. Available at https://green-alliance.org.uk/wp-content/uploads/2021/11/Cutting_climate_impact_of_land_use.pdf (accessed 6 December 2022).

[CIT0028] Green Alliance (2022) The opportunities of agri-carbon markets: policy and practice. Available at https://green-alliance.org.uk/wp-content/uploads/2022/01/The_opportunities_of_agri-carbon_markets.pdf (accessed 6 December 2022).

[CIT0029] Hansson, A., Haikola, S., Fridahl, M., Yanda, P., Mabhuye, E. and Pauline, N. (2021) Biochar as multi-purpose sustainable technology: experiences from projects in Tanzania, *Environment, Development and Sustainability*, 23, pp. 5182–5214. doi:10.1007/s10668-020-00809-8

[CIT0030] Harvey, F. (2009) Black is the new green, *Financial Times*, February 28.

[CIT0031] Harvey, F. (2022) Truthful climate reporting shifts viewpoints, but only briefly, study finds, *The Guardian*, June 20.

[CIT0032] Helliwell, R. and Burton, R. J. F. (2021) The promised land? Exploring the future visions and narrative silences of cellular agriculture in news and industry media, *Journal of Rural Studies*, 84, pp. 180–191. doi:10.1016/j.jrurstud.2021.04.002

[CIT0033] Houses of Parliament (2010) POSTNOTE: Biochar. Available at https://www.parliament.uk/globalassets/documents/post/postpn358-biochar.pdf (accessed 6 December 2022).

[CIT0035] Innovative Farmers (2020) Final report: Biochar for soil and livestock health. Available at https://www.innovativefarmers.org/field-lab?id=0a0868eb-8fe1-e711-816a-005056ad0bd4 (accessed 6 December 2022).

[CIT0036] International Biochar Initiative (2022) IBI webinar: Verified carbon removal by smallholder farmers. Available at https://www.youtube.com/watch?v=sovT57cvgJ4 (accessed 17 June 2022).

[CIT0037] IPCC (2018) Special report: Global warming of 1.5°C. Available at https://www.ipcc.ch/sr15/ (accessed 16 June 2022).

[CIT0038] Jaspal, R. (2020) Content analysis, thematic analysis and discourse analysis, in: G. Breakwell, D. Wright, and J. Barnett (Eds) *Research Methods in Psychology*, pp. 285–312, 5th ed. (London: Sage).

[CIT0039] Karaosmanoǧlu, F., Işigigür-Ergüdenler, A. and Sever, A. (2000) Biochar from the straw-stalk of rapeseed plant, *Energy and Fuels*, 14(2), pp. 336–339. doi:10.1021/ef9901138

[CIT0040] Kleiner, K. (2009) The bright prospect of biochar, *Nature Climate Change*, 3, pp. 72–74.

[CIT0041] Lakoff, G. and Johnson, M. (1980) *Metaphors We Live By* (Chicago, IL: Chicago University Press).

[CIT0042] Latawiec, A. E., Królczyk, J. B., Kuboń, M., Szwedziak, K., Drosik, A., Polańczyk, E., Kuppusamy, S., Thavamani, P., Megharaj, M., Venkateswarlu, K. and Naidu, R. (2016) Agronomic and remedial benefits and risks of applying biochar to soil: Current knowledge and future research directions, *Environment International*, 87, pp. 1–12. 26638014 10.1016/j.envint.2015.10.018

[CIT0043] Leach, M., Fairhead, J. and Fraser, J. (2012) Green grabs and biochar: Revaluing African soils and farming in the new carbon economy, *The Journal of Peasant Studies*, 39(2), pp. 285–307. doi:10.1080/03066150.2012.658042

[CIT0044] Leech, G. (2006) *Glossary of English Grammar* (Edinburgh: Edinburgh University Press).

[CIT0045] Leendertz, L. (2013) Biochar: A slow-burn success, *The Daily Telegraph*, April 6.

[CIT0047] Luokkanen, M., Huttunen, S. and Hildén, M. (2014) Geoengineering, news media and metaphors: Framing the controversial, *Public Understanding of Science*, 23(8), pp. 966–981. 23825283 10.1177/0963662513475966

[CIT0048] Maccoby Berglof, A. (2011) The long-lasting, eco-friendly, carbon-storing wonder stuff, *Financial Times*, May 21.

[CIT0049] Macnaghten, P. and Chilvers, J. (2014) The future of science governance: Publics, policies, practices, *Environment and Planning C: Government and Policy*, 32, pp. 530–548. doi:10.1068/c1245j

[CIT0050] Markusson, N. (2022) Natural carbon removal as technology, *WIREs Climate Change*, 13(2), pp. 1–16. doi:10.1002/wcc.767

[CIT0051] Miller, T. (2012) Letter: Nuclear is not the only option, *The Guardian*, May 25.

[CIT1002] Millar, N., Batalo, B. and Budgell, B. (2022). Trends in the use of promotional language (hype) in abstracts of successful national institutes of health grant applications, 1985-2020. *JAMA Network Open*, 5(8), pp.e2228676–e2228676. 36006644 10.1001/jamanetworkopen.2022.28676 PMC9412227

[CIT0052] Monbiot, G. (2009) Woodchips with everything. It’s the Atkins plan of the low-carbon world, *The Guardian*, March 24.

[CIT0053] Murray, J. (2019) This dark material: The black alchemy that can arrest carbon emissions, *The Guardian*, November 29.

[CIT0054] Nelkin, D. and Lindee, M. S. (2004) *The DNA Mystique: The Gene as a Cultural Icon* (Ann Arbor: University of Michigan Press).

[CIT0055] Nelson, S. C., Yu, J. H. and Ceccarelli, L. (2015) How metaphors about the genome constrain CRISPR metaphors: Separating the “text” from its “editor”, *The American Journal of Bioethics*, 15(12), pp. 60–62. 10.1080/15265161.2015.1103815 PMC4790449 26632368

[CIT0056] Nerlich, B. and Jaspal, R. (2012) Metaphors we die by? Geoengineering, metaphors, and the argument from catastrophe, *Metaphor and Symbol*, 27(2), pp. 131–147. doi:10.1080/10926488.2012.665795

[CIT0057] Nerlich, B. and Jaspal, R. (2013) UK media representations of carbon capture and storage: Actors, frames and metaphors, *Metaphor and the Social World*, 3(1), pp. 35–53. doi:10.1075/msw.3.1.02ner

[CIT0058] Nerlich, B., Evans, V. and Koteyko, N. (2011) *Low carbon diet*: Reducing the complexities of climate change to human scale, *Language and Cognition*, 3(1), pp. 45–82. doi:10.1515/langcog.2011.003

[CIT0059] NFU (2021) Our journey to net zero: Farming’s 2040 goal. Available at https://www.nfuonline.com/media/rwzkb3fc/our-journey-to-net-zero-2021.pdf (accessed 2 December 2022).

[CIT0060] Nicholas, H. L., Halfacree, K. H. and Mabbett, I. (2022) Public perceptions of faecal sludge biochar and biosolids use in agriculture, *Sustainability*, 14, p. 15385. doi:10.3390/su142215385

[CIT0061] Osaka, S., Bellamy, R. and Castree, N. (2021) Framing ‘nature-based’ solutions to climate change, *WIREs Climate Change*, 12(5), pp. 1–20. doi:10.1002/wcc.729

[CIT0062] Otte, P. P. and Vik, J. (2017) Biochar systems: Developing a socio-technical system framework for biochar production in Norway, *Technology in Society*, 51, pp. 34–45. doi:10.1016/j.techsoc.2017.07.004

[CIT0063] *Oxford English Dictionary*. online. OED.com.

[CIT0065] *Oxford Learner’s Dictionary*. online. Hyperbole. https://www.oxfordlearnersdictionaries.com/definition/english/hyperbole.

[CIT0066] Porter, K. E. and Hulme, M. (2013) The emergence of the geoengineering debate in the UK print media: A frame analysis, *The Geographical Journal*, 179(4), pp. 342–355. doi:10.1177/10.1111/geoj.12003

[CIT0067] Pourhashem, G., Hung, S. Y., Medlock, K. B. and Masiello, C. A. (2019) Policy support for biochar: Review and recommendations, *GCB Bioenergy*, 11, pp. 364–380. doi:10.1111/gcbb.12582

[CIT0068] Pragglejaz Group. (2007) MIP: A method for identifying metaphorically used words in discourse, *Metaphor and Symbol*, 22(1), pp. 1–39.

[CIT0069] Price, C. (2021) The online genetically modified food debate: Digital food activism, science and alternative knowledges, *Digital Geography and Society*, 2, pp. 1–10. doi:10.1016/j.diggeo.2021.100017

[CIT0070] Rice-Oxley, M. (2019) The Uside’s best things that happened in 2019, *The Guardian*, December 13.

[CIT0071] Rittl, T. F., Arts, B. and Kuyper, T. W. (2015) Biochar: An emerging policy arrangement in Brazil?, *Environmental Science and Policy*, 51, pp. 45–55. doi:10.1016/j.envsci.2015.03.010

[CIT0072] Roberts, K. G., Gloy, B. A., Joseph, S., Scott, N. R. and Lehmann, J. (2010) Life cycle assessment of biochar systems: Estimating the energetic, economic, and climate change potential, *Environmental Science and Technology*, 44(2), pp. 827–833. doi:10.1021/es902266r 20030368

[CIT0073] Royal Society and Royal Academy of Engineering (2018) Greenhouse gas removal. Available at https://royalsociety.org/-/media/policy/projects/greenhouse-gas-removal/royal-society-greenhouse-gas-removal-report-2018.pdf (accessed 2 December 2022).

[CIT0074] Sams, C. (2009) Biochar is a good tool for climate mitigation, *The Guardian*, September 8.

[CIT0075] Saxe, J. P., Boman, J. H., Bondi, M., Norton, U., Righetti, T. K., Rony, A. H. and Sajjadi, B. (2019) Just or bust? Energy justice and the impacts of siting solar pyrolysis biochar production facilities, *Energy Research and Social Science*, 58, pp. 1–12. doi:10.1016/j.erss.2019.101259

[CIT0077] Sharma-Sindhar, P. (2014) Cool planet: Can biochar fertilise soil and help fight climate change? *The Guardian*, September 2.

[CIT0078] Schenuit, F., Colvin, R., Fridahl, M., McMullin, B., Reisinger, A., Sanchez, D. L., Smith, S. M., Torvanger, A., Wreford, A. and Geden, O. (2021) Carbon dioxide removal policy in the making: assessing developments in 9 OECD cases, *Frontiers in Climate*, 3, p. 638805.

[CIT0079] Soentgen, J., Hilbert, K., Groote-Bidlingmaier, C. v., Herzog-Schröder, G., Pabst, E. E. and Timpf, S. (2017) Terra preta de índio: Commodification and mythification of the Amazonian Dark Earths, *Gaia*, 26(2), pp. 136–143. doi:10.14512/gaia.26.2.18

[CIT0080] Summerley, V. (2012) In the black: Craig Sams, the man behind Whole Earth and Green & Black’s is getting his hands dirty with an organic charcoal invention. Victoria Summerley invites him into her garden to put his new venture to the test. *The Independent*, June 22.

[CIT0081] Sweet, S. K., Schuldt, J. P., Lehmann, J., Bossio, D. A. and Woolf, D. (2021) Perceptions of naturalness predict US public support for soil carbon storage as a climate solution, *Climatic Change*, 166(22), pp. 1–15. doi:10.1007/s10584-021-03121-0

[CIT0082] TerrAffix (2022) TerrAffix: Soil-habitat-carbon. Available at https://terraffix.co.uk/ (accessed 6 December 2022).

[CIT1001] *The Daily Telegraph* (2013) Biochar: A slow burn success, *The Daily Telegraph*, April 8.

[CIT0083] *The Daily Telegraph* (2021) Radio choice, *The Daily Telegraph*, September 2.

[CIT0084] *The Guardian* (2009a) National: Six climate plans, *The Guardian*, July 4.

[CIT0085] *The Guardian* (2009b) Turning charcoal into carbon gold, *The Guardian*, August 27.

[CIT0086] *The Guardian* (2009c) Locking up carbon with biochar, *The Guardian*, July 13.

[CIT0087] *The Guardian* (2009d) Can cloud-making ships, giant algae ‘stomachs’ and the lessons of the Serengeti save us? *The Guardian*, July 5.

[CIT0088] *The Independent* (2009a) Letters, *The Independent*, January 6.

[CIT0089] *The Independent* (2009b) His dark materials: The man behind Green & Black’s chocolate wants to save the planet with charcoal, *The Independent*, September 27.

[CIT0090] Thornhill, J. (2021) Climatetech 2.0 must sell venture capital on its future, *Financial Times*, October 21.

[CIT0091] Vaughan, A. (2022) Farmers in England will bury burnt wood in fields to capture CO2, *New Scientist*, May 24.

[CIT0092] Walker, J. (2012) Peat-based compost is bad for the environment, but are greener alternatives any good?, *The Guardian*, June 16.

[CIT0093] Wright, M. J., Teagle, D. A. H. and Feetham, P. M. (2014) A quantitative evaluation of the public response to climate engineering, *Nature Climate Change*, 4(2), pp. 106–110. doi:10.1038/nclimate2087

[CIT0094] Wyatt, S. (2004) Danger! Metaphors at work in economics, geophysiology, and the Internet, *Science, Technology, & Human Values*, 29(2), pp. 242–261.

[CIT0095] Zimmer, J. (n.d.) Rhetorical devices: Tricolon. Available at https://mannerofspeaking.org/2015/03/16/rhetorical-devices-tricolon/ (accessed 16 June 2022).

